# Thinking with attachments: Appreciating a generative analytic

**DOI:** 10.1177/03063127211048804

**Published:** 2021-10-06

**Authors:** Marianne de Laet, Annelieke Driessen, Else Vogel

**Affiliations:** 1 Harvey Mudd College, Claremont, CA, USA; 2 University of Amsterdam, Amsterdam, The Netherlands; 3 London School of Tropical Hygiene and Medicine, London, UK

**Keywords:** attachments, feminist STS, actor-network theory, care, material semiotics

## Abstract

Much current work in Science and Technology Studies inflects knowing with care. Analyses of the ethos of objectivity, and of the practices by which objectivity is crafted, have shown that knowing and caring cannot be thought apart from each other. Using case studies from our own work we analyse how, in the sociotechnical relationships that we study, knowing and caring are entangled through ‘attachments’. We appreciate – both in the sense of valuing or respecting and in the sense of evaluating or assessing – how the notion of ‘attachment’ invites re-imagining relations between the social and the technical, between knowers and objects known, and between sociotechnical work and the affective sensibilities that enable, and are brought to life by, such work. Our respective ethnographic engagements with dog-human relations, obesity surgery and dementia care demonstrate that it is agents’ diverse and shifting attachments to technologies and techniques that shape the ways in which bodies, knowledge and practices form. The affects that arise in this process, or so we claim in neo-pragmatist fashion, are not preconditions to, but rather the result of such practices of attachment; rather than a prerequisite, they are an effect of the work of attaching itself. Thinking with attachments recognizes how techno-scientific work builds and shapes passions, aesthetics and sensory experience, allowing us to trace how varied sensibilities to what constitutes ‘the good’ come to be and come to matter in practices of relating between humans, animals and things.

Feminist epistemologies, ethnographic susceptibilities and praxiographical approaches to ontology have prompted scholars in Science and Technology Studies (STS) to take seriously bodies and mundane processes, to recognize the implications of their materialities and respect their fickle infidelities. As bodies come with quirks, materials with relations, and practices with uncertainties, these sensibilities bring under fresh scrutiny the ways in which connections within networks form and hold. As we address not only how things hang together but also how they fall apart, it is no longer sufficient to imagine connectivity in terms of forces, vectors, powers and bonds of greater or lesser strength. Such seemingly neutral concepts, signalling mechanics and calculatory logics, are belied by scholars’ and citizen scientists’ explorations of care, tinkering, intimacy and tacit knowing in laboratories, medicine and other science-related, day-to-day practices.^1^

Calling out the ways in which the care that infuses such practices has historically been made invisible – pushed out of knowledge-making regimes and professional public life, and into the domain of the private, the domestic and the somatic (Latimer and López Gómez, 2019; Mol et al., 2010) – feminist STS scholars, in particular, have complicated ‘the pervasive bifurcations that prioritize the rational over the sensory and affective dimensions of knowledge’, by highlighting ‘the moral and affective economies that shape researchers’ entanglements with the phenomena they describe’ (Martin et al., 2015: 7). In other words, in the making of knowledge, care and normativities count.

*How* they are *made* to count matters. Speaking in terms of moral and affective ‘economies’, for instance, attributes a structure to the moral and affective realms, suggesting that a logic is at work here. Moral and affective compulsions that inflect scientific and technical work are not, or so this narrative goes, untamed, intuitive, ‘natural’, individual – and therefore disruptive – sensitivities. They rather form a web of sensibilities crafted in social practices and – however implicitly – are bound to a regime of rules and shared understandings that are endemic to, and are productive of, such work. Challenging the notion that emotions are private – ‘belonging’ to the individual – this seemingly paradoxical term, ‘affective economies’, suggests that emotions are not private ‘things’ located ‘within’; they are instead aspects of the entanglements of agents, in particular circumstances. But these affects help produce the logic of the autonomous and emotion-less scholarly body, while also bringing into existence the very configuration of the world ‘surrounding’ it as a space encompassing insides, outsides, surfaces and boundaries – and, in the process, shaping the normative commitments that keep all of these notions alive.

If the feminist STS tradition has recognized that scholarly work and the knowing body are *not* neutral, that, as researchers, we are normatively engaged and have skin in the game, in this paper we are curious about the life of such productive normativities in the practical engagements that research entails. Not only is it important to appreciate that norms and values shape all kinds of inquiry; we have also learned that the practices of inquiry shape norms, values and passions, in turn. A language is called for, then, which thinks about such relations differently (Latimer and López Gómez, 2019), which eschews bifurcations, and instead enables the complications of the moral economies of research practices to come into view. In this paper we try out the term ‘attachments’, borrowed from (a subset of) actor-network approaches, as a heuristic to capture such relations. Thinking with attachments, or so we hope to show, helps account for the shape-shifting, meandering, loose, flexible, sensitive and temporary connections that make socio-technical configurations stick, vacillate, regroup and disassemble. We argue that this notion of ‘attachment’, with its corollary of ‘detachment’, adds a much-needed specificity to relations between the social and the technical, between knowers and objects known and between techno-scientific work and the affective sensibilities that enable – and are brought to life by – such work.

Our framing matters. Where ‘thinking *about* attachments’ would force a separation between our minds and the object under consideration, our effort to ‘think *with* attachments’ signifies that, in line with Verran’s (2017) suggestion, we treat our ‘concepts as companions’. Attachment is not a phenomenon ‘out there’ for us to study, but a sensitivity that opens up (or indeed, attaches us to) particular worlds and forms of world-making, while closing down others. ‘Thinking with’, then, is an invitation to explore the potential of attachments as an analytical instrument – but also to seek out its limits.

## From the cellars of ANT

Over the past twenty years, since it started to circulate in the vaults of the Centre *de Sociologie de l’Innovation* (CSI) in Paris, the term ‘attachments’ has come to signal the work done by bonds, affects and auxiliaries in the complex and not necessarily articulated moves that fold technologies, facts and artefacts into the day-to-day (Gomart and Hennion, 1999; Hennion, 2001, 2017; Teil and Hennion, 2004).^2^ As such, attachments draw attention to coincidence and circumstance. They are achievements – or so CSI sociologist Antoine Hennion and others suggest – that arise from sometimes planned, more often emergent, engagements with others and things (Hennion, 2007a, 2007b). They are specific to a locale and its material devices, are done by bodies that are learning to sense and that have the agency to act as well as surrender, and rely on collaborations of sorts with objects that give (feed)back as they are tried, tested, tasted, put into use, crafted or falling in disrepair. In the process of attaching bodies, relationships form while they are also already in play: They are co-constituted as and when attachment unfolds. The term ‘attachments’ adds a tool to the growing and shifting STS repertoire, prompting us to explore the material and affective forms that connections take through valuing, appreciating, liking and disliking. Such probing of the valences of connections invites attending to the trajectories between stability and decay, push and pull, force and counterforce, attraction and disinterest that render networks into more than grids with nodes.

As a way into such relations, attachments continue to live within the CSI tradition of doing STS. In actor-network fashion, rather than taking on scientific knowing and the artefacts of technoscience, attachments inform observations of the mundane practices in which knowers and the objects of knowledge are co-construed. Music, drugs, wine – these are examples of ‘objects’ of appreciation that emerge while knowing and those that know are simultaneously in the making (Ávilá Torres, 2016; Callén Moreu and López Gómez, 2019; Gomart and Hennion, 1999; Mishra, 2020; Teil, 1998, 2012). Of such ‘making’, attachments are a crucial part: As serious, framing, effective (in the sense of ‘having an effect’) entanglements between humans and non-humans, attachments are constitutive of ecologies of knowledge. And if this literature on attachments deliberately orients itself towards unfolding sensibilities in the practices of lay persons, it picks up a concern within STS with the mundane procedures, situated performances and distributed expertise that mark expert knowing, as they do that of ‘amateurs’.

Perhaps its structural resonance with the recursive play of ANT imaginings has impeded attachment, as an analytical term, from traveling far beyond the SCI and French neo-pragmatist circles. Indicating ‘the co-creation of affinities, things, bodily engagements, and collectives that develops when a person or a group comes to strongly like [something]’ (D’Hoop, 2018), attachments rely on agents’ capacity to ‘be affected’. The very idea of attachment, then, flies in the face of the parameters by which knowledge and knowing bodies are framed. Originally proposed, according to Hennion, as an antidote to linear models of society-technology interactions where agency is staged as a centralized and active faculty of humans (and humans alone), the term attachment signals an ANT concern with the making of divides between the social and the technical, the human and the non-human, the affective and the objective realms. Highlighting not only the productive role of objects in such interactions, through its focus on mediation the term points out ‘forms of agency beyond the active/passive dualism’ (Hennion and Muecke, 2016: 300). If dismissed by some as an apolitical or amoral actor-network inspired attempt to distribute agency among humans and non-humans without attention to their moral and affective differences, we suggest that it is *precisely* for its political capacity to reframe relations between technology and society, knowledge and care, making and knowing, action and agency, objects and subjects, experts and amateurs, and humans, animals and things, that the conversation about attachments is worth taking to heart. It is, we submit, exactly for its attention to the effect of relating in the indeterminate and shape-shifting workings of ‘the good’ that the term holds analytic and political promise.

## Attachments and associations: A material-semiotic landscape

In the foreword to Despret’s (2016) collection of ‘scientific fables’, *What Would Animals Say If We Asked The Right Questions?*, Latour (2016) names Despret an ‘additive’ rather than a ‘subtractive’ empirical philosopher. While ‘interested in objective facts and grounded claims’, as she studies the world of animal studies research, she likes ‘to add’, according to Latour, ‘to complicate, to specify, and, whenever possible, to slow down, and … hesitate so as to multiply the voices that can be heard’. In other words, she attends to the *attachments* that form (in) the human-animal engagements that she reviews. Instead of critiquing dismissively or placing ideologically the (animal) science that her book is about, Despret probes its performative work, turns upside down its outcomes and premises and, by reframing, re-values what it has to offer – all the while minding the manifold and multivalent attachments between the animal scientists, amateurs and animals whose relationships are at stake.

Despret’s (2016) review of the work of ethologist Lorenz is a point in case. While ethological and other scientific practices typically rely on the notion that subject and object – here, human and animal under observation – are fixed, stable and separate entities, conventional representations of Lorenz’s ideas – according to Despret – systematically overlook the entanglements between the two.^3^ The relational event is a matter of ontology, she suggests, rather than epistemology (or ideology): As the ethologist ‘studies’ the Jackdaw the bird becomes ‘Jackdaw-with-Human’ as much as Lorenz becomes the reverse. Complicating further the well-known ethological story of how the scientist gets to know the bird, she argues that his knowledge is only possible as the bird gets to know the ethologist.^4^ Suggesting – and here the interest in the origins of the bifurcations shows up again – that the distinction between subject and object is the analyst’s preoccupation rather than the participants’ matter of concern, Despret argues that knowledge, object and subject are co-produced in a complicated process of mutual shaping through what we call attachment. In learning to be *affected by* each other, both researcher and bird morph into something else and it is the investigation *itself* that mediates between, and so binds and shapes, the two. Even if many scientific practices cultivate a *de*tachment between researcher and researched, such detachment is not the opposite or absence of relation, but a relation in itself (Candea, 2010). Being, as Whitehead (1920/2004) suggests, can only be in relating; the relating is instantaneous and cannot be undone, because the inquiry both produces *and* relies on the other-in-relation-to-self.^5^ Here, then, in its affected, hesitant, instant, inescapable and complex with-ness-ing (Haraway, 2008), we see attachment concretely at work.

The acknowledgement that entities and their (normative) positions become through the attachments of which they are a part turns thinking with attachments into a distinctive sociology, reworking ontologies of the social. Whereas in a classical Durkheimian sociology, social organization overlays the material world, in a framework that centres attachments, this world and its organization emerge in concert with one another. Stratifying society according to an *a priori* set of variables such as gender, class, religion, age and race, conventional sociology explains constituents’ preferences in reference to these categories. Using this logic, a sociologist might, for instance, discern a firm connection between class and musical preference: If folks from the upper-middle class attend the opera, they are playing out a distinguishing identity game in which loving opera is by definition an upper-middle class trait; it indexes upper-middle-classness (see Bourdieu, 1984). Determining structures such as ‘culture’ and ‘society’ are so mobilized to explain cultural and social forms. A hallmark of actor-network derived approaches is to call out the tautology in such explanations, and to reverse the causal arrows between ‘actors’ and ‘the social’. The representation produces the object, rather than the other way around (Callon, 1984; Latour, 1987; Latour and Woolgar, 1986; Woolgar, 1988). As we have seen, thinking with attachments is indebted to actor-network approaches for this neo-pragmatist impulse to reverse cause and effect.

The mechanism that makes this reversal plausible in an ANT frame is the ‘association’ (in the literature sometimes used synonymously with ‘attachment’), which can be understood as any linkage of any sort. So, in a complementary set of early ANT papers tracing the connections between technologies and power differentials in 16th and 18th century colonial relations, Law (1986) and Latour (1986) explain how technologies of travel, navigation, collection and categorization *establish* and *perform* (rather than reflect) power inequities. It is not power that makes empire ‘hang together’; rather, power ensues from the socio-material relationships enabled by empire. Published around the same time, and developed in collaboration with Law, Latour, Hennion and others at the CSI, Callon’s (1984) famously symmetric treatment of scallops, fishermen and scientists mobilizes the term ‘association’ to analyse the socio-technical system at hand. Describing the efforts of marine researchers to avert the decline of scallops in St Brieuc Bay as a series of opportunistic connections, Callon distributes agency equally among humans, scallops and things. The paper’s conceptual repertoire frames the scientists’ relations with fishermen and scallops as a series of stakeholder investments, an interest-driven, rather mechanistic endeavour (Star, 1990).

While the actor-network question of how relations enact social cohesion (rather than the reverse) has begun to inform STS theory at large, a focus on attachments places this question more explicitly at centre-stage. What if we were to think through Callon’s case as a making, unmaking and remaking of attachments? We might then say that, rather than the scientists failing to enroll the other actors in St Brieuc Bay, neither party has a firm grasp on how to relate, yet all clamour to engage their counterparts. It is the frivolity of the attachments in play, and the *de*tachments that represent actors’ diverging passions, concerns and dependencies, that undermine the coherence of the system, so accounting for an impasse in which neither the problem of a declining scallops population, nor a solution for this problem, can reliably be shared by all. More importantly, neither problem nor solution is a solid state: As agents shift position, so do their attachments, and as a result what seemed an impasse yesterday may turn into opportunity tomorrow – and vice versa. Retroactively inserting attachments in Callon’s work is not critique; arguably, it is thanks to this work that we can mobilize the concept today. This exercise rather suggests the relational attachments between the terms themselves, invoking the lively theoretical space in which they co-exist with associated concepts and, in circulation, crystallize, take hold and leave the scene when they are done.

In what follows, we develop moments from our own research to explore the additive value of thinking with attachments in connection with tried and tested STS themes, such as the ontology of the social, (nonhuman) agency and the work of the senses in knowledge practices. To the material-semiotic sensibility in the ANT tradition, which understands all things in the natural or social world as continuously generated effects of the web of relations in which they are located (Law, 2007), thinking with attachments adds an attention to the workings of sensing and care. Recognizing that this mode of thinking does not live in a vacuum, we have roughly mapped the material-semiotic landscape in which attachments live. In Despret’s additive spirit, we do not mean for the term attachment to *replace* other analytical terms such as association (Callon, 1984), entanglement (Barad, 2010; Latimer and López Gómez, 2019) or care (Mol, 2008). Nor is it meant to *correct* other relational STS concepts such as translation, domestication or co-production. It instead *adds* a heuristic to the STS repertoire.

## Relating reflexively

Within this landscape, our own work attends to various forms of care, which we understand as a set of reflexive relationships: shifting sensibilities, sensitivities, concerns and interventions that the work of attachments holds together. It is not sufficient to recognize that norms and values inform analysis; our proposal to think with attachments implies that the practices of our analyses shape the norms, values and passions that we carry forth. Taking attachments seriously, we must acknowledge that entities, including researchers and their normative positions, *are becoming* through the attachments of which they are a part. Thinking with attachments thus explicitly queries the politics of knowledge production and, in so doing, revives the reflexive impulse in STS work.

That our analytic practices have such effects is perhaps what Martin et al. (2015: 11) mean when they suggest that normativity is among ‘the very conditions of possibility for care’. We wonder, however, whether the caring attitude of the researcher – ‘a person who cares must first be willing and available *to be moved by* this other’ (Martin et al., 2015) – may inadvertently rely on a liberal notion of agency and a human-centred notion of care. Speaking in terms of attachments highlights, more than anything, that as researchers and people we do not *just* or *only* enter into a relation with others as a matter of will. If ‘object’ and ‘subject’ infect and inflect each other, becoming – through the attachments they form – blended figures that each include the other, then focusing on attachments serves to describe a process of care that entails the capacity of being moved *inadvertently* – and not only by humans, but by other agents as well. This process relies on making oneself available in the broadest sense – responsive and response-able – which is in turn made available by a bestiary of socio-material elements and agents at work.

The political power of thinking with attachments, then, lies in recognizing the play of stability and flux, in its invitation to observe the elements that are in play, sort through their relative qualities, valences and forms, and attend to the normative effects of specific attachments in the particular and temporal circumstances of the setting at hand. If care is characterized by practical negotiation and tinkering between heterogeneous ‘goods’ and ‘bads’ (Mol et al., 2010), then attachment refers to the embodied processes of qualification and valuing that animate such practices; it demands care for the premises, normativities and unarticulated but productive assumptions that infuse not only the practices of care but also those of care studies. In this way, or so we suggest, thinking with attachments asks us to observe the work of relating that constitutes the landscape of our research engagements and, in doing so, contributes to an emerging attention to being-as-relating as a salient theme in our field. As thinking with attachments begins to inform ethnographic work in technology, medicine, care and mundane practices involving knowing and technoscience, it inspires taking account of bodies, affectivity, valuation and normative dispositions in ways that eschew pitting the rational against the affective. Observing attachments in action and at work so calls attention to the shifting contours of the research space as it develops. More saliently, perhaps, it calls for an account of how one’s own normative and intellectual commitments build in concert with the attachments that one forms as a researcher, an observer and a participant in that space.

## Lead or leash? Ontology of a social

What holds things together? Togetherness is the question rather than the premise of sociologies of association (Callon, 1984; Latour, 2005) and attachment (Gomart and Hennion, 1999). Both frameworks for understanding socio-technical relations take as their point of departure a social in-the-making and unknown. Social stabilities are wrought and strange; permanence is the exception, rather than the norm and it is a temporal effect of the research engagement itself. Such efforts to develop a non-tautological approach to the social – a social that includes power, empire and explicitly the ‘human mind’ – do not imagine this social as a set of determining relationships but rather as their result and they bring the work of relating squarely into view. Understood not as a separate metaphysical sphere, nor as a known set of realities, but as a shape-shifting network that emerges as humans and nonhumans connect – or get attached – the social is a work in progress of which there is no end in sight.^6^ This work crafts coherence, and it includes efforts of maintenance and care. These are heterogeneous efforts that have to be made again and again, lest things come undone. Instead of taking the ‘social whole’ as what explains such ‘holding together’, it is precisely the sociologist’s presumption of togetherness that needs accounting for.

In its effort to account for togetherness, an approach that attends to attachments adds a concern for preferences, passions, effort, agencies and aesthetics in a way that working with associations might not quite allow. Foregrounding attachments acknowledges the material and differential mediations that produce the effect of being (or allowing oneself to be) moved. While associations demonstrate *that* relating matters, and care engages the question of *how* it matters, attachments invite attending to how relating to and between humans, animals and other things *comes* to matter. Thus, when Cochoy et al. (2017) describe the crafting of a market as the work of attachments, they frame the activity of exchange not so much as a platform for investments, but as the result of associations, negotiations and calculations about profit and risk; its objects not as pre-existing but rather as produced by this market itself. It is attachments, not just links – between humans and each other, but also such things as products, produce, money, double entry bookkeeping and gold – that bring together the social phenomenon that is called a market. As Callon (2017) puts it in the same volume, ‘the process of attachment is … a process of expression, in which [agents] learn what they are and what they are becoming, and whereby, symmetrically, things and goods express what they are or what they can do and “make do”’ (p. 181). Relations matter, and they are the stuff of which materials are made. But the reverse is also true: Materials matter, and they are the stuff of which relations are made.

In her current work De Laet (2021) considers the work of forming a pack with her animals, attending to the attachments that form, but also *per*form, human and dogs as they walk together on a lead.^7^ Or do they walk together on a leash? While the tether between human and dog, in English, can be described by either term, dog afficionados tend to prefer ‘lead’ over ‘leash’ – to indicate that the human may lead, but does not have the dog in bondage; the word can also be taken to mean that, in leading, human and dogs may take turns. Complicating the valuation of the leash, while it is typically understood as the machine that subjects the dog, it can also suggest the human’s subservience to the animals in her care. As participant observer in and of the machinations of the pack in which she is both an insider and outsider, de Laet asks how, in this relationship between the dogs and their human, the material object that is the lead or the leash helps form and stabilize sensibilities, sensitivities, norms and togetherness. While the *leash* is undeniably an instrument of power, it is not entirely clear who is leashed by whom. And understood as a *lead*, the tether is *also* a conduit of sensitivities: a language that expresses connectivity, a medium through which the agency of both human and dog can express itself, an object through which and to which attachments are forged, expressed, enabled and understood, or a relationship through which subjects are forged. Assisted by the lead/leash, human and dogs learn about each other – and, together, learn to form a multiheaded new beast. De Laet must do the walk, as both the dogs and her sanity demand it, and the leash enables, shapes, signals, signifies and produces it, all at the same time. While the lead doesn’t necessarily make for a good walk – as its subjects may try to gallop off in all directions – the leash *may* keep all of them on a straight path. And while the lead may put the dogs (or a dog) in charge, walking on a leash might teach both human and dogs how to walk well and be well together.

If the leash suggests that leadership during a walk resides with the human subject, it more often than not shifts it to one or all of the dogs. They pause, they sniff, they equivocate, they circle back, inviting their human to follow them – as they know best what is interesting about the terrain. They are attached with and without the lead, though – if they persist in hanging back, their human can drop the leash and even without being tethered they will follow. As it coordinates the pack, the leash also crafts it; without it, another subject – that of humans and dogs freely attached – shows up. The pack, its individual subjects, and its blended ones, take many forms and every move of attaching creates new subject positions that subsume old ones without altogether dissolving them. Goods and bads are in play here: A disobedient or stubborn dog is bad, but so is a hectoring or impatient human; a dog happily running around freely, as long as her human is nearby and ‘in control’, is good – but so (for some) is a human who is firmly attached to a dog. Depending on the day, the moods, the weather and what transpired before, a walk can be a breeze or it can be a struggle, and either can be good or bad. Dogs leading can be pleasant or annoying; a leading human can guide the dog (good) or cruelly subdue her (bad). As it takes shape, shapes and re-shapes the pack, the walk must negotiate attachments and hold in the balance all of these valences and qualifications. It is a momentous and consequential event.

The walk, then, is an achievement that is crafted through attachments but at the same time forges them. The leash/lead is not only a tether – attachment, materialized – or a link that associates human and dogs, but, in addition, an object through which new and shifting subjectivities come into effect. While through this tether attachment is practiced and formed, over time, in collaboration and resistance, and with agencies distributed among human, dogs and material object, such attachment is never stable, always shifting, and renegotiated through the presence of the material tie.

How do things hold together? De Laet suggests that togetherness gels by attending to one another’s presence, that such togetherness is not always smooth, and that it requires continuous work. It needs to be done again and again, at each walk, at each visit, at each interaction, at each moment that one of the constituents takes the lead. In the intricacies of going on a walk, agency and control diffuse among actors; words, leash/lead, human and dogs at work craft fidelity to the practice of being a pack. If attachment may be framed as an emergence or refinement of sensibilities in the practices of the mutual, shared, but diverging connections between the dogs and their human – all, arguably, irremediable amateurs in and of each other’s worlds^8^ – the sensibilities that shape themselves are indeed the product and the conditions of the intense focus on living together that living together *well* demands. In the process of the leashed walk, human and dogs become framed in light of being with the other; it is from the walk that the agents ‘dog-with-human’ and ‘human-with dog’ – as well as the knowings and attachments that frame these agents – spring.

What emerges here is a sensibility that attends to the work of keeping materiality and relationship in connection with each other. And in this paradoxical complex of the material and the relational, a moment of wonder presents itself: How, in shifting worlds and environments, with conflicting stakes and interests, do ‘things’ ‘hold’ ‘together’? Rather than taking for granted the stability of worlds and words and things, this wonder orients us towards querying the mechanisms that make (for) coherence, stability and boundedness – however fleeting or fragile the resulting imbroglios may be.

## Attaching to guts: Refiguring agency

In Gomart and Hennion’s (1999) sociology of attachments, attachment is framed as a ‘mode of doing’. Exploring the practices of music amateurs and drug users, Gomart and Hennion argue that in the day-to-day engagements with their habits, practitioners subject themselves to what objects (music, instruments, drugs, technique) do to and with them. Meanwhile, in a recursive move, their activities *produce* the properties of these objects that act so strongly upon them. Surrendering oneself is thus not a passive act: Allowing oneself to be affected emerges as a generative, active practice. Borrowing Callon’s line, the authors call out this blurring of activity and passivity as *faire-faire*: ‘being left to and made to arrive’ (Gomart and Hennion, 1999) or, as Latour (1999) translates it, ‘to make (one) do’ or ‘causing to be done’. A second STS theme – next to ontologies of the social – where thinking with attachments makes an intervention, then, concerns the nature of agency.

In similar fashion, Struhkamp et al. (2004) complicate agency in medical practice. Suffering, normally framed as a passive subjection, emerges here as actively and collectively shaped by participants in medical rehabilitation practices. Picking up the Dutch term *laten*, which means ‘to leave’ or ‘to let go’, the authors argue that *not to do* commands agency, too. Requiring listening, not-solving and articulating, *laten* is an act.^9^ Tracing attachments – and detachments – so opens up an inquiry of what it ‘is’ to act. It is a way of studying agency that foregoes the question of who or what does the acting in favour of describing the particularities and world-making effects of diverse modes of doing. Such effects may include the exhilarating qualities of music or drugs (Gomart and Hennion, 1999), the enactments of powerful managerial actors (Callon and Law, 2005; Latour, 1988; Law, 1994), or the human-dog pack configuring itself around the leash. In any case, recognizing that there is agency at work in ‘opening oneself to being affected’ nuances the conventional wisdom that agency implies mastery or control, that doing is doing something *to* another. In thinking with attachments, our understanding of agency shifts: There is agency in giving over as much as there is in being in control. Action is refigured so as to escape active-passive, agent-structure, free-determined or subject-object binaries.

Such sensibilities are at work in Vogel’s (2018) inquiry into care practices to do with obesity prevention and treatment. At first sight, the idea of obesity surgery, a set of procedures in which the digestive system is rearranged to achieve reduced nutrient uptake and thus ensure weight loss, might suggest that the surgeon takes over the management of obesity, intervening from the outside onto a passive patient. A closer look at the attachments, practices and normativities at play in one obesity clinic in the Netherlands leads Vogel to argue that people diagnosed as ‘morbidly obese’ actually take on the difficult task of becoming ‘active subjects through submission and (re-)attachment and by arranging support’ (Vogel, 2018: 510).

Obesity surgery – which doctors currently consider the only path to long-lasting substantial weight loss – affords a mode of embodiment that brings patient’s cravings in line with each other, by ‘anchoring’ the will in the body’s anatomy. While the procedure thus holds great promise for patients who have struggled with what some of them have been used to calling ‘their’ obesity, often for quite some time, it is disruptive, too. Intestinal and digestive problems, disordered eating, addiction and malnutrition are not uncommon in the wake of surgical intervention. Clinicians further warn that without a change in lifestyle, the weight loss for which the patient goes to such great length might not last. In the clinic of Vogel’s fieldwork an ‘empowerment lifestyle program’ helps patients engage in healthy activities, manage complications, and deal with the emotional and social consequences of bodily change. For as the surgically altered ‘stomach’ may complicate ingrained practices of self-care through eating, patients must learn to anticipate how it may interfere with social eating patterns and food preferences. Such getting in sync with the body ‘emerges as a matter of aligning, adjusting, attending, organizing and letting go. One change invites another, and in the process, patients do not only *act* differently, they (their feelings, inclinations and appreciations) come to *be* different’ (Vogel, 2018: 520).

As patients intervene in their own anatomy, they do not do so all by themselves. Agency comes to closely align with support and submission, as patients actively rely on others to craft forceful changes on themselves. Rather than *as an actor* who is in control and free, the body that emerges through these practices *is enacted* through the relations of which it is a part. Normative questions abound: What are better, rather than worse ways of becoming together? Attachments, then, do away with a tendency in the social sciences to favour autonomy out of fear of domination, bringing renewed political curiosity towards the conditions for what Gomart (2002) calls *generous constraint*: Forces that capture an entity ‘without destroying it’ (p. 521). The normative effort in this analysis, in Latour’s (1999) words, is not to ‘distinguish between the restrained and the liberated, but instead between the well and the poorly attached’ (p. 23).

Although the sites of her fieldwork are clearly marked by strong medical, aesthetic and gendered norms around a good body, good food and good behaviour, Vogel hesitates to analyse her informants’ efforts as evidence of how, struggling to adhere to norms handed down to them, they simply ‘internalize’ these norms. Instead, she highlights how patients are embroiled in a constant effort to attach, and to attach well. Through the work of getting in synch with the body, ‘good’ and ‘bad’ attachments present themselves, ‘both within the skin and outside of it’ (Vogel, 2018: 519). With the help of clinicians, patients pragmatically sort these attachments – even if (and exactly because!) through this sorting, they completely transform. Thinking with attachments, then, affords prominence to practices of reflexivity: A constant testing, assessing, calibrating appreciation is at the heart of participants’ engagement with their environment. It is central, too, in the practice of theorizing these practices: By articulating the normativities at play and the tensions between them, scholars contribute to shaping the fragile, situated ideal of ‘good care’ (Mol et al., 2010). Studies of care practices and other socio-technical entanglements may refuse to judge but nevertheless are committed to probing ways to live well with/in attachments. That commitment places norms inside, rather than outside, the work of science, technology and care; it prompts the question not only of how norms govern actions but, also and more importantly, how they are crafted as knowledge, practices and techniques are made.

## Dementia matters: Sense, sensitivity, sensibility

Although predicated on the ability to be affected, attachments should not be confused with affects. Our concern here is not with attachment as ‘lasting psychological connectedness between human beings’ (Bowlby, 1969: 194) – a psychological phenomenon that inscribes love and affection as basic developmental needs.^10^ Understood in psychology as an (inter-)personal dynamic that operates ‘on’ or ‘in’ relationships, its paradigmatic case is the apparent connection between parent and child. In contrast, we do not offer a ‘theory of attachment’ here, nor do we frame attachment as psychological force. Rather, as we discuss the work of attachment in techno-social configurations, the term is a heuristic, a theoretical tool. The body of work in STS that brings attachments into focus, always rooted in specific case studies, invites telling stories about knowing, doing, caring and crafting that probe the processes, the bodies, the objects, the subjects and the practices that, *while in the making*, are at work. The term attachment, we suggest, shifts the modes of accounting from telling how people and things relate and what the world looks like, to stories of how people, things and worlds *emerge* – however fragile, temporary, fluid or flexible these worlds may be.

Neither should attachment be confused with an innate sensitivity. The ability to ‘sense’ is not a faculty one ‘has’; sensing is rather a practice from which the senses develop. Rather than an individual and innate capability, sensing emerges as the crafted effect of the relations between worlds and their bodies. Describing her efforts to become an expert *nez* [‘nose’] in the perfume industry, Teil (1998) argues that smell is not ‘just’ an object that noses register (or fail to register); it is a practice (also Latour, 2004). Elsewhere, in a study of wine tasting, she argues that it is through the practice of tasting itself that differences in wine flavours come into existence. Tasting is not a faculty – but, rather, a technique, technology and material performance all at once. In practitioners’ developing attachments to the gestures, environments, materials, fellow-tasters, words, aromas and other elements of the acts of tasting something that may be called ‘taste’ is forged. The wine and its flavours are not the cause but rather the effect of these collective tasting activities, routines, conversations and other interventions that tasters make in their own and others’ appreciative process. Similarly, Alač’s ethnography of an olfactory psychophysics laboratory reveals smelling as a crafted sensibility. As smelling turns out to be neither uniquely private and in the subject’s control, nor entirely public, the ‘subject’ cannot be regarded as the location of a sense; rather, subjects emerge from their sensing immersion in the world (Alač, 2020a, 2020b). Another one of Despret’s stories is in order here. Retelling the famous account of Hans, the horse who could count, and his caretaker-researcher who incites him to, Despret describes the ongoing work of crafting a pair of mutually sensitive bodies. Hans, in Despret’s rendering, does something much more interesting than math; he enables the humans questioning him to develop sensitivities and sensibilities that without him would remain unarticulated and unexplored. In their interaction, the humans gain a body that does more things, that feels other events, and that is more and more able to lead [Hans]’ (Despret, 2004). Through their attachment, each party learns to be moved by the other; ‘[w]ho influences and who is influenced … are questions that can no longer receive a clear answer. Both, human and horse, are cause and effect of each other’s movements. Both induce and are induced, affect and are affected’ (Despret, 2004: 115).^11^

The senses are thus pertinent to – as they are subject to – the process of becoming-in-relating. With that established, curiosity arises about how sensitive worlds are made. If one is an ‘excellent nose’, a ‘discerning taster’, an ‘average smeller’, one is becoming so in a complex set of engagements. No longer is taste a natural faculty, sensitivity a ‘given’ characteristic of a stable body or a feature determined by the class, schooling and educative sophistication into which that body is socialized. Taste does not reside in the qualities of either object or subject – it is a product of the activity in which subject and object become ‘together’: collaboratively, in tension or in collusion, simultaneously involved. Sensibility and sensitivity develop; they ‘are’ not, but are made (Mann, 2015, 2018). And such moving and making requires effective and affective work.

Taking this very starting point, in her ethnography of Dutch nursing homes for people with dementia, Driessen (2018) brings out the collective character of crafting sense and sensitivities – at once. Public imaginary of dementia stages the condition as a neurological pathology that inevitably leads to a passive life with no hope of joy. Indeed, portrayals of nursing homes report on elderly residents ‘dozing’, placidly watching television; suggesting a causal, necessary and inescapable connection between dementia and apathy. Thinking with attachments allows a different version of dementia; it counters the idea that pathology blocks affect and that – thus! – in the absence of pathology, affect is a given.

Taking up Gomart and Hennion’s (1999) claim that ‘active work must be done in order to be moved’, Driessen narrates care practitioners’ efforts for the nursing home’s residents’ affects. Care emerges as the conduit that enables them to take pleasure in dancing, bathing, music and movement. Everyday tasks emphasize such pleasures: In one field site a care worker and a physical therapist organize dance events, in another, staff reshuffles tasks and schedules to repurpose an underused bathroom to allow for one-on-one moments in which residents receive personal, spa-like attention. In providing bodily care through unconventional elements such as music, drinks and togetherness, care professionals bring about attachments and so, Driessen (2018) argues, ‘craft conditions for pleasure’ (p. 28).

Importantly, she notes, enjoyment cannot be forced. Attachments to these new elements of care do not always form, and residents are not mere recipients. For as they interact with these things that may or may not afford pleasure, a play of invitation and acceptance affords staff and residents a chance to develop sensitivities – to one another and to the things around them. And in this play, rather than singularly catering to the sensory capacities that ‘still are’, more fully sensing residents emerge: ‘Music entices the body to move. The care professional or volunteer extends a hand and the stability for a dance … the resident accepts the hand, steps onto the dance floor, moves along and leads’ (Driessen, 2018: 29). Invited into such attachments, residents who accept can come to feel enjoyment – and, surprisingly, so may others. For when residents enjoy themselves, bystanders do so as well. ‘If the care workers let themselves be affected, they too emerge as joyful subjects’ (Driessen, 2018: 29). While affect emerges here from the work done for people with dementia, the case raises a more general point, that speaks to those with and without cognitive difficulties alike: Sense and sensitivity are not given and are therefore not irrevocably lost; they do not come in predictable form but rather come about through collective investments in attachments.

As ‘[a]ppreciations may be missed by those not receptive to them’ (Driessen, 2018: 34), thinking with attachments – or so we suggest – has consequences for the practice of knowledge production in STS itself. Calling on ethnographers to learn to be sensitive, to extend an invitation to their interlocutors to enact their subjectivity and, so, to enable unforeseen potentialities, Driessen (2018) suggests that making responsive and responsible knowledge indeed demands from the researcher a willingness and capacity to be moved (pp. 34–35). But – and this is what is distinctive about this approach – ‘attachments do not *belong* to people nor do they *define* them: Depending on situations, forging their existence and history through debates and confrontations, they have to be continuously done and re-done. They both appear as a constraint and a resource to people confronted by destabilizing problems’ (Hennion, 2017: 118). If the senses do not precede the world, but are enticed, incited, developed or attuned in their relations to it, *what* is known is not separate from *how* it is known; affected by sensitivities and sensibilities, knowing also produces them. Attending to attachments means attending to looping experiments in which humans and things/animals/technologies are in the process of (re-)shaping each other. Thinking with attachments, then, attends to mediations and effects, realizing that, and bringing into view how, bodies, subjects, tastes, objects, communities and appreciations are produced, and come to matter, together.

## Conclusion

We have described attachment as a generative sensibility that is specific to, and constitutive of, subjects in practice. Such practice involves materials, bodies, gestures, collectives, built environments, situations and locales. Thinking with attachments, as we have shown, allows the articulation of the social as more than an *a priori* domain or a conflict of interests; agency beyond active/passive and liberal/deterministic dualisms; and sense and sensibility beyond a taken for granted set of bodily affects. If ways of knowing, practices and techniques are interlaced with and shaped by attachments, they are different than the ones we are used to attending to in STS. For attachments give rise to complex and shifting normativities; they cannot be adjudicated or assessed by norms that rule from elsewhere. And so it is modes of attending to, and shifting attachments to, techniques, knowings and modes of doing, that come into view. At stake here is exploring normativities as they unfold and shift in this work of attending, appreciating and attaching, and probing attachments as ways of bringing out, experimenting with and doing the good. Attach*ment* involves trials, effort, shifting, shaping, doing. Attach*ing* is a reflexive, normative practice, a way to act, while holding close the question of what it is to act well.

We propose to think *with* attachment to ask anew how things temporarily hold together, or remain apart, and how they become, evolve and perish. Technologies, techniques, knowledge and practices – yes. But, having been primed by our work on attachments to note instability, we ask how these manage to act, how they come in relation, how they shape, shift and form. In the face of crumbling polities, eroding environments, risk-full engagements, unstable compounds, impermanent bodies and structures that fall apart, such questions are all the more pertinent. If, collectively, ‘we’ do not excel at building enduring and stable connectivities, then the instances of living together, moments of care, and examples of things ‘holding’ that point to the contrary matter all the more.

We share this commitment with others in feminist STS who have attended to care in science, technology and beyond. If scholarly work is not neutral, but normative and engaged, how should we understand this normativity? Today, care and affect are not only considered as empirical topics of study, but also, in a reflexive move, as an invitation to question the politics of our own knowledge production (de la Bellacasa, 2017). Following Felski’s (2015) exploration of the limits of distant, sceptical critique, we can now understand being political or ‘normative’ in our work differently. Taking seriously de la Bellacasa’s (2011) call ‘to exhibit the concerns that attach and hold together matters of fact is to enrich and affirm their reality by adding further articulations’ we are interested in *assembling* rather than disarticulating relations. Here, too, thinking with attachment spurs a conversation about what worlds we help enact, strengthen or make invisible as we study and write, and prompts us to reflect on what might be better rather than worse forms of world-making.

All too often feminist ethical-political commitments are themselves recast in affective terms: Some scholars claim that when engaging the worlds we study, ‘we cannot but care’ (Martin et al., 2015). We argue, however, it is not just important to recognize that norms and values shape all kinds of inquiry. Our proposal to think with attachments underscores that our very practices of inquiry shape our principles, beliefs and passions, too. What is ‘right’ and ‘wrong’ is not given but made, emerging through our engagements with a situated field. In other words, taking attachments seriously, we must acknowledge that entities, including the researcher, *are becoming* through the attachments of which they are a part. Affect is not the precondition but, on occasion, a *result* of practices of knowing and caring – emerging in concert with other entities. And making oneself available is *made available* by other socio-material elements that are in play. The political potentialities of thinking with attachments lie in the analytical work of listing, sorting through, and attending to valences and normativities of the attachments that shape the research practice at hand.^12^

Thinking with attachments means attending to what Pols (2015) calls ‘intranormativity’: To a careful and mindful way of attending to local, situated, distributed and emerging goods and bads. So, thinking the leash with attachments does not draw us immediately into the hierarchical politics of animal husbandry and power, but takes up such concerns in order to, instead, open up a sensitivity to the social and material intricacies of living together (well). The case of obesity surgery, when thought with attachments, does not call out directly the (gendered) establishment of body norms and forms of bodily control, but invites a conversation of what constitutes ‘good care’ in the face of the conflicting set of physical, psychological and social challenges and norms that coalesce around obesity. Thinking dementia with attachments, finally, reimagines dementia as more than just a neurological condition characterized by loss. Instead, it emphasizes how specific relations surface response-ably in the lives of people with dementia and those who care for them, generating senses and sensitivities.

These situated analyses reveal that where there are bads, there are also goods; where things hold, they also crumble. ‘Attachments’, then, account for how things stick; detachments, for how they come apart. Attending to *how* things emerge as good or bad and to *what* solidifies or disintegrates when passions, concerns or desires are pointed towards some good, they form a missing link in accounting for sociotechnical worlds. As valuing is a productive act, enacting entities through engagement, it makes worlds happen. Foregrounding the co-production of bodies and practices – as fickle but firm, present but in formation, emerging in and as knowing, affected but not driven by affect – thinking with attachments not only highlights *that* and *how* affect matters, but also how it *comes* to matter. So tracing the shifting relationships that in probing ontologies of the social we must take into account our stories, or so we think, make for critical yet hopeful analysis. Being normative, then, is not simply taking position ‘for’ or ‘against’; it means opening up to being moved by the situated normativities of the worlds one inhabits as researcher. Inspired, still, again, by Teil’s, Latour’s and Hennion’s work on the doing of taste, by Despret’s investigations of animal engagements, and by feminist suspicions of the modes of objectivity, we see attachments as among the things that mediate in, and with, technologies to accomplish such worlds. And so this paper is an invitation *to* and, or so we hope, is itself an instrument *for* becoming affected by attachments. As all theoretical tools, concepts have limits. But theoretical fluidity is a strength of STS. In an additive spirit, and without becoming too attached, we encourage you, dear reader, to think with attachments, to explore them – and toss them or add yet other tools if and when they turn out to burden more than they afford.

## Notes

1 The list of inspirations here is long but let us highlight Coopmans and McNamara (2020), Friese (2019), Haraway (2008), Jespersen et al. (2014), Latimer (2013), Martin et al. (2015), Mol et al. (2010), Pinel et al. (2020), Pols (2014, 2017), de la Bellacasa (2017) and Skeide (2019).

2 Inspired by James (1912/1976) pragmatism and Whitehead (1929/1978) process theory, this conversation also resonates with similar framings developed in STS-related fields elsewhere – for instance with the work of Haraway (2008) and others who push material-semiotic sensibilities.

3 If and when Konrad Lorenz’s work is discussed in a relational sense, it is to point out his membership of the German Nazi party in the 1930s and the pernicious connections between his ethological contributions and the Nazi ideology that he at the time endorsed. While Despret does not address pressing concerns about Lorenz’s Nazi sympathies, to questions of how ideological commitments and science inform each other she adds that subject and object – and, as we submit, by extension ideology and science – re-shape each other in their interaction. The implications of Despret’s analysis are worth exploring; perhaps, as a result of their engagement, neither Nazi doctrine nor Lorenz’s ethological commitments can or should be imagined separate from each other.

4 See also Candea’s (2010) ‘I fell in love with Markus the Meerkat’. In contrast to our emphasis on attachments, Candea shows how scientists in their knowledge practices care for *de*tachment, arguing that detachment is not the opposite or absence of relation, but a relation in itself.

5 For the relevance of being-as-relating to STS see Haraway (2008), Latour (2005) and Stengers (2011).

6 The work of Gabriel Tarde is an inspiration here (also, Candea, 2015; Latour, 2002).

7 For a discussion of distributed ownership see also Woolgar and Neyland’s (2013) chapter 7 in *Mundane Governance*, which features ‘baggage and its passenger’, and Hache’s (2015) paper, ‘If I have a dog, my dog has a human’.

8 For more on being amateurs in the worlds of others, see Despret’s (2016) chapter ‘U is for Umwelt which digests Jakob von Uexkuell’s theory of *Umwelt* for the purposes of STS.

9 Note the implicit pushback here, to a heroic, gendered, problem solving mode of action that is – at least in occidental philosophy – generally assumed to be the only way to be active (see, e.g. Jullien, 1995 for a contrast between Western and Chinese frameworks for efficacy in the latter of which effective action includes ways of letting go).

10 The paradigmatic research here being Harry Harlow and colleagues’ research on mother-child bonds in monkeys (Harlow et al., 1965). Tragically, while this research established the importance of love and closeness, it is anchored in the abusive practice of withholding affection to the animals involved.

11 Schrader (2015) notes that ‘becoming affected’ is a temporal process; how, when and in what order we ‘begin to care’ about something – in her case, how and when one begins to care for deformed leaf bugs after the Chernobyl disaster – determines what it is one cares about.

12 See also Bruun Jensen (2007) for an exploration of the value of normative approaches in STS, and Ballestero (2019) as she explores the performative effects of listing, categorizing, indices and other types of administrative record-making.
